# Real-world osimertinib pretreatment experience in patients with epidermal growth factor receptor T790M mutation-positive locally advanced or metastatic non-small cell lung cancer

**DOI:** 10.1371/journal.pone.0303046

**Published:** 2024-05-16

**Authors:** Gee-Chen Chang, Jin-Yuan Shih, Chong-Jen Yu, Heng-Sheng Chao, Cheng-Ta Yang, Chien-Chung Lin, Jen-Yu Hung, Sheng-Yen Hsiao, Chin-Chou Wang, Chih-Feng Chian, Te-Chun Hsia, Yuh-Min Chen

**Affiliations:** 1 Department of Internal Medicine, Division of Pulmonary Medicine, Chung Shan Medical University Hospital, Taichung, Taiwan; 2 School of Medicine and Institute of Medicine, Chung Shan Medical University, Taichung, Taiwan; 3 Department of Internal Medicine, Division of Chest Medicine, Taichung Veterans General Hospital, Taichung, Taiwan; 4 Department of Internal Medicine, National Taiwan University Hospital, Taipei, Taiwan; 5 National Taiwan University, Taipei, Taiwan; 6 Department of Internal Medicine, National Taiwan University Hospital Hsin-Chu Branch, Hsin-Chu, Taiwan; 7 College of Medicine, National Taiwan University, Taipei, Taiwan; 8 Department of Chest Medicine, Taipei Veterans General Hospital, Taipei, Taiwan; 9 School of Medicine, National Yang Ming Chiao Tung University, Taipei, Taiwan; 10 Department of Internal Medicine, Division of Thoracic Medicine, Chang Gung Memorial Hospital at Linkou, Taoyuan, Taiwan; 11 College of Medicine, Chang Gung University, Taoyuan, Taiwan; 12 Department of Internal Medicine, National Cheng Kung University Hospital, Tainan, Taiwan; 13 College of Medicine, National Cheng Kung University, Tainan, Taiwan; 14 Department of Internal Medicine, Kaohsiung Municipal Ta-Tung Hospital, Kaohsiung, Taiwan; 15 Department of Internal Medicine, Division of Hematology-Oncology, Chi Mei Medical Center, Liouying, Tainan, Taiwan; 16 Department of Medicine, Division of Pulmonary and Critical Care Medicine, Chang Gung Memorial Hospital-Kaohsiung Medical Center, Kaohsiung, Taiwan; 17 Department of Internal Medicine, Division of Pulmonary and Critical Care Medicine, Tri-Service General Hospital, Taipei, Taiwan; 18 National Defense Medical Center, Taipei, Taiwan; 19 Department of Internal Medicine, China Medical University Hospital, Taichung, Taiwan; 20 Department of Respiratory Therapy, China Medical University, Taichung, Taiwan; National Institute of Cancer Research, TAIWAN

## Abstract

Osimertinib has demonstrated efficacy in patients with epidermal growth factor receptor (EGFR) T790M-positive non-small cell lung cancer (NSCLC) in clinical trials. However, real-world data on its effectiveness remain scarce. Taiwanese patients with T790M-positive locally advanced or metastatic NSCLC and progressive disease following treatment with at least one EGFR tyrosine kinase inhibitor (TKI) were enrolled from the osimertinib early access program. Of the 419 patients (mean age, 63 years; female, 67%), 53% were heavily pretreated (≥ third-line [3L]), making osimertinib a fourth-line (4L) intervention. The median progression-free survival (PFS) was 10.5 months (95% confidence interval [CI]: 8.95–11.41); the 18-month PFS rate was 26.5%. The median overall survival (OS) was 19.0 months (95% CI: 16.30–20.95); the 24-month OS rate was 40.9%. The objective response rate was 32.46%, and the disease control rate was 86.38%. The median time to treatment discontinuation of osimertinib monotherapy was 11.9 months (95% CI: 10.49–13.11). Subgroup analyses of median PFS and OS in the chemotherapy combination group vs. the osimertinib monotherapy group yielded no difference. Central nervous system (CNS) metastasis, number of prior lines of therapy, and types of initial EGFR-TKIs did not significantly impact outcomes. The median PFS values were 9.0 (95% CI: 5.18–11.34) and 10.9 (95% CI: 9.18–11.90) months with and without CNS metastasis, respectively, and 10.8 (95% CI: 8.59–12.69), 13.6 (95% CI: 10.89–16.3), and 9.2 (95% CI: 7.8–10.62) months for second-line (2L), 3L, and ≥4L therapy, respectively. In patients who received osimertinib as 2L therapy, the median PFS values in response to prior afatinib, erlotinib and gefitinib treatment were 11.2 (95% CI: 4.85–4.79), 10.5 (95% CI: 8.59–20.26) and 8.7 (95% CI: 7.21–16.79) months, respectively. Overall, real-world data from Taiwan support the clinical benefits of osimertinib in EGFR T790M -positive NSCLC.

## Introduction

Lung cancer is the leading cause of cancer death in Taiwan and remains a global health concern [1]. The most common type of this cancer is non-small cell lung cancer (NSCLC), which accounts for 80–90% of lung cancers [2]. Effective treatment of NSCLC has been limited due to activating epidermal growth factor receptor (EGFR) mutations. Studies examining EGFR mutation frequency revealed a much higher rate of 51.4% among Asian populations compared to 10–20% in North American and European populations [3–5].

Despite advanced molecular profiling established to direct the use of targeted therapy such as tyrosine kinase inhibitors (TKIs), acquired resistance—most often driven by the T790M mutation—is well documented in patients who were treated with first-generation and second-generation frontline EGFR-TKIs [5, 6]. The combination of platinum-based therapy and pemetrexed has been widely used in patients with centrally confirmed EGFR T790M-positive advanced NSCLC demonstrating progressive disease (PD) after first-line (1L) EGFR-TKI therapy [7, 8]. However, response rates have been only in the 20–30% range [9–11]. The results from the AURA clinical trials on the efficacy and safety of osimertinib—a third-generation, central nervous system (CNS)-active EGFR-TKI that specifically targets T790M-mediated resistance—demonstrated a high objective response rate (ORR) and disease control rate (DCR) [12–14]. Pooled data analysis of phase II AURA extension and AURA2 studies demonstrated an ORR of 66% and DCR of 91% [15]. In the AURA phase 3 study comparing platinum-based doublet therapy with osimertinib, the median progression-free survival (PFS) was significantly longer with osimertinib than with platinum-based therapy plus pemetrexed (10.1 vs. 4.4 months) [16]. Similarly, the ORR was significantly higher with osimertinib than with platinum-based therapy plus pemetrexed (71% vs. 31%). The proportion of patients with grade >3 adverse events (AEs) was lower with osimertinib (23%) than with platinum-based therapy plus pemetrexed (47%) [16].

These findings have led investigators to further examine the use of osimertinib in real-life heterogeneous patient populations outside of the clinical trial setting. Results from real-world studies conducted in Japan, Germany, and France have demonstrated high response rates among all subgroups [17–20]. The ORR and DCR of a Japanese real-world study were 60.7% and 91.1%, respectively, closely paralleling results from the AURA2 pooled analysis [15, 18]. The German early access program (EAP) reported similar ORR and DCR values of 80.0% and 95.9%, respectively [19]. ASTRIS, an international real-world study examining the effectiveness and safety of osimertinib, also reported investigator-assessed clinical response rates of 57.1%. The median PFS and median time to treatment discontinuation (TTD) were 11.1 and 13.5 months, respectively [21]. The overall survival (OS) data are still immature at the time of publication and will be reported in future studies. With the largest real-world sample to date (*n* = 3015), the results from ASTRIS support the clinical benefits of osimertinib in pretreated patients with EGFR T790M-positive advanced NSCLC without any new safety signals of concern.

In Taiwan, osimertinib EAP was approved by the Taiwan Food and Drug Administration for the treatment of EGFR T790M-positive locally advanced or metastatic NSCLC from October 2015 to September 2016. Here, we present the clinical data from the Taiwan EAP on the real-world effectiveness of osimertinib administration in patients with EGFR T790M-positive locally advanced or metastatic NSCLC following progression on at least one prior EGFR-TKI treatment.

## Materials and methods

### Study design

This work is a retrospective observational study of patients from 10 medical centers across Taiwan diagnosed with EGFR T790M-positive locally advanced or metastatic NSCLC. Prior to its approval on 10 November 2016 in Taiwan, osimertinib was made available through an EAP to patients who demonstrated PD following 1L EGFR-TKI therapy. Data were collected from the medical charts of EAP patients who met the following eligibility criteria for inclusion: 1) at least 20 years of age and 2) received at least one dose of osimertinib monotherapy. Patients who received osimertinib as 1L treatment were excluded from this study. Osimertinib was orally administered at a dosage of 80 mg once daily. All patients in the EAP received the first dose of osimertinib prior to October 2016 and had the potential for at least two years of follow-up. Informed consent was obtained from all participants enrolled in the study according to local institutional review board (IRB) and independent ethics committee (IEC) regulations. Ethical approval for this study was obtained from the IRB/IEC of the respective participating facilities.

### Data collection

Data on baseline patient demographics and clinical characteristics were collected from the medical charts of enrolled subjects and included age, sex, smoking status, body weight, height, and Eastern Cooperative Oncology Group Performance Status (ECOG-PS) prior to the first dose. In addition, tumor-associated characteristics were extracted, including tumor stage and histology, tumor site, presence of metastases and/or other malignancies, and comorbidity burden. Details of tumor stage and site at two additional time points were gathered—prior to receiving the first NSCLC systemic therapy and immediately before the initiation of osimertinib. Investigators assessed tumor response at the initiation of the first dose of osimertinib, at the time of the first disease progression while on osimertinib monotherapy, and at the point of its discontinuation. The best tumor response was determined during the period from the first dose of osimertinib to the date of PD, discontinuation, or death, whichever occurred first. Information on EGFR mutation testing and status other than T790M as well as other NSCLC treatment history prior to receiving the first dose of osimertinib were also harnessed.

### Study endpoints

The primary endpoint was PFS, defined as the time interval from the date of the first dose of osimertinib to the date of PD according to the investigators’ assessments or to the date of death regardless of cause, whichever came first.

Secondary endpoints included 1) OS, defined as the time interval from the first dose of osimertinib until the date of death due to any cause, 2) TTD, defined as the time interval from the first dose of osimertinib until the date of discontinuation of osimertinib monotherapy for any reason including disease progression, drug-related AEs, or death, 3) prior NSCLC treatment, 4) rationale for use of osimertinib over other NSCLC treatments, 5) sample types and test platforms used to confirm acquired EGFR T790M mutation and the status of other types of EGFR mutation as applicable, 6) subsequent lines of systemic NSCLC treatment with osimertinib monotherapy, and 7) reasons behind the investigators’ prescription of such add-on therapy. Investigator-assessed tumor responses were categorized into complete response (CR), partial response (PR), stable disease (SD), PD, or not determined (ND). PFS and OS data were further stratified by subgroups to compare patients with and without CNS metastasis, subsequent chemotherapy after discontinuation of osimertinib monotherapy, and different initial EGFR-TKIs prior to second-line osimertinib treatment.

### Statistical analysis

Descriptive statistics were used to analyze patient characteristics and recorded variables. Kaplan‒Meier methods were applied to estimate PFS, OS, and TTD. The estimated PFS rate at 12 and 18 months and OS rate at 18 and 24 months were also analyzed. The full analysis set (FAS) was defined as all EAP participants who received at least one dose of osimertinib.

## Results

### Baseline demographics and clinical characteristics

A total of 423 patients (mean age, 63 years; female, 67%) were included in the FAS. Among those whose ECOG-PS score was known, patients with ECOG-PS ≥2 comprised approximately 20% of the total population.. The comorbidity burden was highest in the cardiovascular system (31.21%), followed by the gastrointestinal and endocrine systems (each constituting 13.95%) and the respiratory system (7.33%). Just under 5% of patients had malignancies of various histologies. Specific comorbid conditions under each system are shown in Table 1, along with the other baseline demographic data and clinical characteristics above. Complete details of the T, N, M staging are summarized in S1 Table.

**Table 1:** Baseline demographics and clinical characteristics.

Characteristic	Total (N = 423)
**Age (years)**	
N	423
Mean (SD)	63.3 (11.90)
Median (min, max)	63.0 (32, 95)
**Gender, n (%)**	
Female	284 (67.14%)
Male	139 (32.86%)
**ECOG Performance Status, n (%)**	
0—Normal activity	43 (10.17%)
1—Symptoms, but ambulatory	127 (30.02%)
2—In bed <50% of the time	29 (6.86%)
3—In bed >50% of the time	12 (2.84%)
4–100% bedridden	2 (0.47%)
Unknown	210 (49.64%)
**Tumor Sites, n (%)**	
Lung	213 (50.35%)
Liver	32 (7.57%)
Lymph node	93 (21.99%)
Skin	1 (0.24%)
Bone	111 (26.24%)
Head and neck	1 (0.24%)
Adrenal	7 (1.65%)
Brain	80 (18.91%)
Pleura effusion/seeding	51 (12.06%)
Other^a^	10 (2.36%)
**Type of histology**	
Adenocarcinoma	411 (97.16%)
Squamous cell carcinoma	1 (0.24%)
Large cell carcinoma	1 (0.24%)
NSCLC-type unspecified	5 (1.18%)
Unknown	5 (1.18%)
**Tumor staging when administered the first dose of osimertinib**	
Non-metastatic	5 (1.18%)
Metastatic	126 (29.79%)
Unknown	292 (69.03%)
**Previous Lines of Therapy, n (%)**	
1	91 (21.51%)
2	108 (25.53%)
> = 3	224 (52.96%)
**Major Comorbidities, n (%)**	
*Cardiovascular System*	
Hypertension	120 (28.37%)
*Respiratory System*	
Chronic obstructive pulmonary disease	14 (3.31%)
*Gastrointestinal System*	
Hepatitis B	30 (7.09%)
Hepatitis C	5 (1.18%)
*Endocrine System*	
Diabetes mellitus	47 (11.11%)
*Malignancy*	
Colon cancer	1 (0.24%)
Non-Hodgkin’s lymphoma, unspecified	1 (0.24%)
Oral cancer	1 (0.24%)
Prostate cancer	1 (0.24%)
Hepatocellular carcinoma	2 (0.47%)
Nasopharyngeal carcinoma	2 (0.47%)
Breast cancer	4 (0.95%)
Cervical cancer	4 (0.95%)
Thyroid cancer	5 (1.18%)

ECOG, Eastern Cooperative Oncology Group; n, number; SD, standard deviation.

^a^Other: Leptomeningeal metastasis, pericardium, pericardial effusion, ascites, kidney, right supraclavicular fossa nodal metastasis, left cervical lymph node, pericardial, spine and spleen.

All patients were EGFR T790M mutation positive, as confirmed through various biospecimen types and testing platforms, of which tissue biopsy (44.42%) and real-time polymerase chain reaction (44.18%) were the most common (S2 Table). The most prevalent mutations in this cohort were exon 19 deletion (52.72%) and L858R on exon 21 (37.12%). There was no significant difference of survival between the patients confirmed by plasma and tissue specimens (median 16.0 vs. 20.9 months, p = 0.1101). The survival curve of patients by plasma and tissue specimens is depicted in S1 Fig.

### Treatment prior to osimertinib initiation

This cohort was heavily pretreated with first-generation and second-generation EGFR-TKIs, chemotherapy, and systemic therapy (25.53% and 52.96% received 2 and 3 previous lines, respectively). The most common treatments were platinum- or taxane-based therapies (29.79%) and radiation therapy (17.02%) targeting CNS metastasis (Table 2). The primary reason for initiating osimertinib treatment in 94.80% of participants was a lack of efficacy of their last NSCLC therapy regimen.

**Table 2:** Last NSCLC therapy prior to the first dose of osimertinib.

Last NSCLC therapy, n (%)	FAS Population (N = 423)
**EGFR-TKI therapy**	
Afatinib	55 (13%)
Erlotinib	95 (22.46%)
Gefitinib	52 (12.29%)
Other EGFR-TKI therapy	8 (1.89%)
Other 3G EGFR-TKI therapy^a^	5 (1.18%)
**Chemotherapy**	
Platinum- or taxane-based therapy^b^	126 (29.79%)
Other chemotherapy	154 (36.41%)
**Other systemic therapy**	
Bevacizumab	17 (4.02%)
Nivolumab	7 (1.65%)
Pembrolizumab	4 (0.95%)
Crizotinib	1 (0.24%)
Capmatinib (INC280)	1 (0.24%)
Selumetinib	1 (0.24%)
AUY922	1 (0.24%)
Bavituximab	1 (0.24%)
Biotherapy	1 (0.24%)
Ramucirumab	1 (0.24%)
Selumetinib	1 (0.24%)
Sunitinib	1 (0.24%)
Clinical Trial	1 (0.24%)
**Radiation therapy for brain metastasis**	
Radiation alone	72 (17.02%)
Radiation combined with systemic therapy^c^	31 (7.33%)
**Combined therapy**	
EGFR-TKI combined with chemotherapy	10 (2.36%)
EGFR-TKI combined with other systemic therapy	11 (2.60%)
Chemotherapy combined with other systemic therapy	14 (3.31%)

EGFR-TKI, epidermal growth factor receptor sensitizing mutation tyrosine kinase inhibitors; FAS, full analysis set; n, number; N, total number in population; NSCLC, non-small cell lung cancer; 3G, third generation.

^a^Other third-generation EGFR-TKI therapies include nazartinib/EGF816, olmutinib/HM61713, naquotinib and rociletinib.

^b^Platinum-based therapies include carboplatin and cisplatin. Taxane-based therapies include docetaxel, nab-paclitaxel, and paclitaxel. The combination of platinum- or taxane-based therapy with other chemotherapy was included in this category.

^c^Included radiation therapy for brain metastasis combined with EGFR-TKI, chemotherapy, or other systemic therapy.

### Osimertinib effectiveness

#### Study endpoints

**Fig 1:**
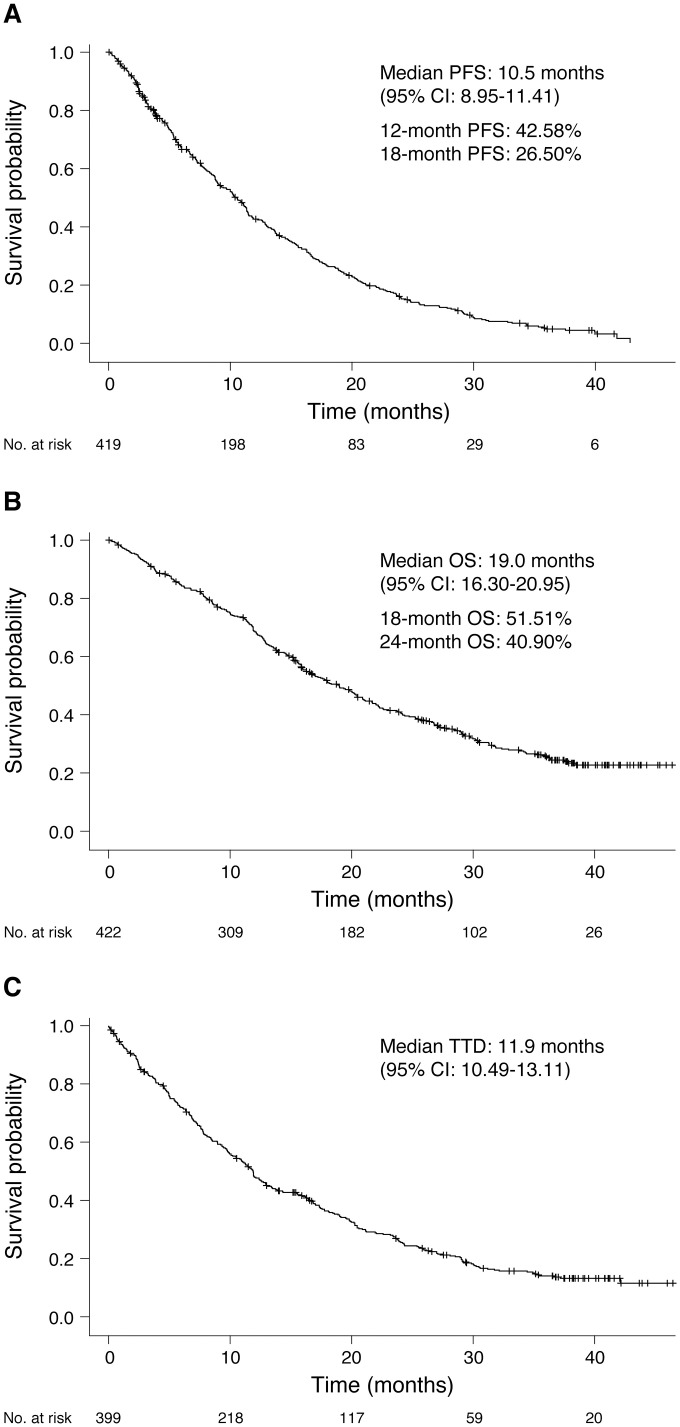
Kaplan‒Meier analysis of (A) PFS, (B) OS, and (C) TTD. CI, confidence interval; OS, overall survival; PFS, progression-free survival; TTD, time to treatment discontinuation.

After excluding 4 patients with either unknown survival status or lack of tumor assessment records, a total of 419 patients were analyzed for PFS. Fig 1A shows the Kaplan‒Meier (KM) estimate of the median PFS at 10.5 months (95% confidence interval [CI]: 8.95–11.41). At 12 and 18 months, the proportions of patients surviving without PD were 42.58% and 26.50%, respectively.

OS data were analyzed for 422 patients (due to one patient being excluded for unknown survival status), of whom 296 (70.14%) had death events during the study period and 126 patients were censored. The median OS was 19.0 months (95% CI: 16.30–20.95), with 18- and 24-month OS rates of 51.51% and 40.90% of patients, respectively (Fig 1B).

Of the 399 patients with available TTD data, 326 (81.70%) discontinued osimertinib monotherapy—primarily due to PD—while 73 (18.30%) participants were censored. The primary reason for the exclusion of 24 patients from the TTD analysis was an unknown date of death or living status. The median time to discontinuation of osimertinib monotherapy was 11.9 months (95% CI: 10.49–13.11) (Fig 1C).

#### Treatment response rates

Clinical outcomes defined by the best tumor response are shown in S3 Table. CR was achieved in 0.58% (*n* = 2) and PR in 31.88% (*n* = 110). More than half of the patients (*n* = 186, 53.91%) were determined to have SD, while 11.30% (*n* = 39) demonstrated PD. The overall ORR was 32.46%, and the DCR was 86.38%. The median PFS by previous lines of treatment was 10.8 (95% CI: 8.59–12.69), 13.6 (95% CI: 10.89–16.30), and 9.2 (95% CI: 7.80–10.62) months with osimertinib as second-line (2L), third-line (3L), and fourth-line (4L) (or more) therapy, respectively (Table 3). The median OS of one vs. two previous lines (osimertinib as 2L vs. 3L) of therapy was 18.0 (95% CI: 15.08–23.05) and 24.2 months (95% CI: 16.43–30.49), respectively (S2 Fig). Previous lines of therapy did not have a significant impact on PFS or OS.

**Table 3:** Median progression-free survival by lines of treatment.

Total (N = 423)		
1 Previous line of therapy		
n	91	
Event number and %	76	83.52%
Median time (in months), 95% CI	10.8	8.59–12.69
2 Previous lines of therapy		
n	108	
Event number and %	83	76.85%
Median time (in months), 95% CI	13.6	10.89–16.30
3 Previous lines of therapy		
n	220	
Event number and %	204	92.73%
Median time (in months), 95% CI	9.2	7.8–10.62

CI, confidence interval; n, number; N, total number in the population.

### Subgroup analyses

#### Subsequent treatments

**Fig 2:**
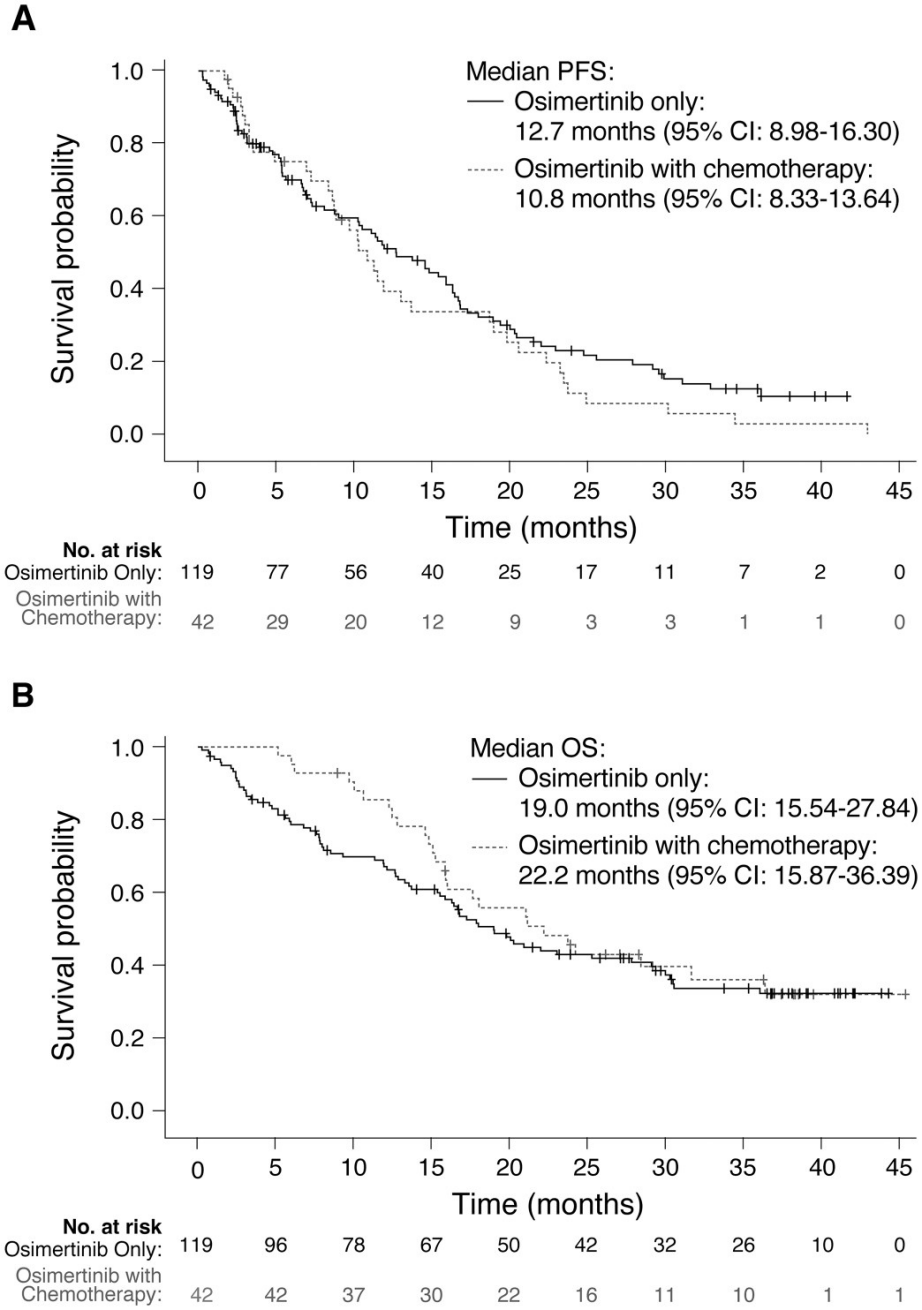
Kaplan‒Meier analysis of (A) PFS and (B) OS by subsequent chemotherapy after osimertinib monotherapy discontinuation. (A) PFS and (B) OS. CI, confidence interval; OS, overall survival; PFS, progression-free survival. Note: Patients who used osimertinib as a second- or third-line therapy are included. The “osimertinib only” group included patients who had osimertinib monotherapy; the “osimertinib with chemotherapy” group included patients who had chemotherapy as the first subsequent systemic regimen combined with osimertinib after discontinuation of osimertinib monotherapy.

S4 Table shows the various subsequent interventions prescribed after the discontinuation of osimertinib monotherapy, stratified by subsequent treatments given in combination with osimertinib vs. treatments given without osimertinib. The primary reason (78.76%) for prescribing other NSCLC treatments as an add-on to osimertinib monotherapy was disease progression. Of the 423 participants, 182 received subsequent systemic treatments after the discontinuation of osimertinib monotherapy. The sum of subsequent systemic treatments exceeded 100% because multiple counting was applied in one participant. Of the 370 subsequent systemic treatments administered, chemotherapy was the most common (289 in total; 164 were combined with osimertinib). Further KM analyses were performed to elucidate the effects of subsequent systemic chemotherapy with osimertinib as a 2L or 3L treatment after osimertinib monotherapy discontinuation (Fig 2A and 2B). No significant differences were found between the osimertinib-chemotherapy combination group and the osimertinib monotherapy group in PFS (10.8 [95% CI: 8.33–13.64] vs. 12.7 [95% CI: 8.98–16.30] months, respectively) and OS (22.2 [95% CI: 15.87–36.39] vs. 19.0 [95% CI: 15.54–27.84] months, respectively).

#### CNS metastasis

**Fig 3:**
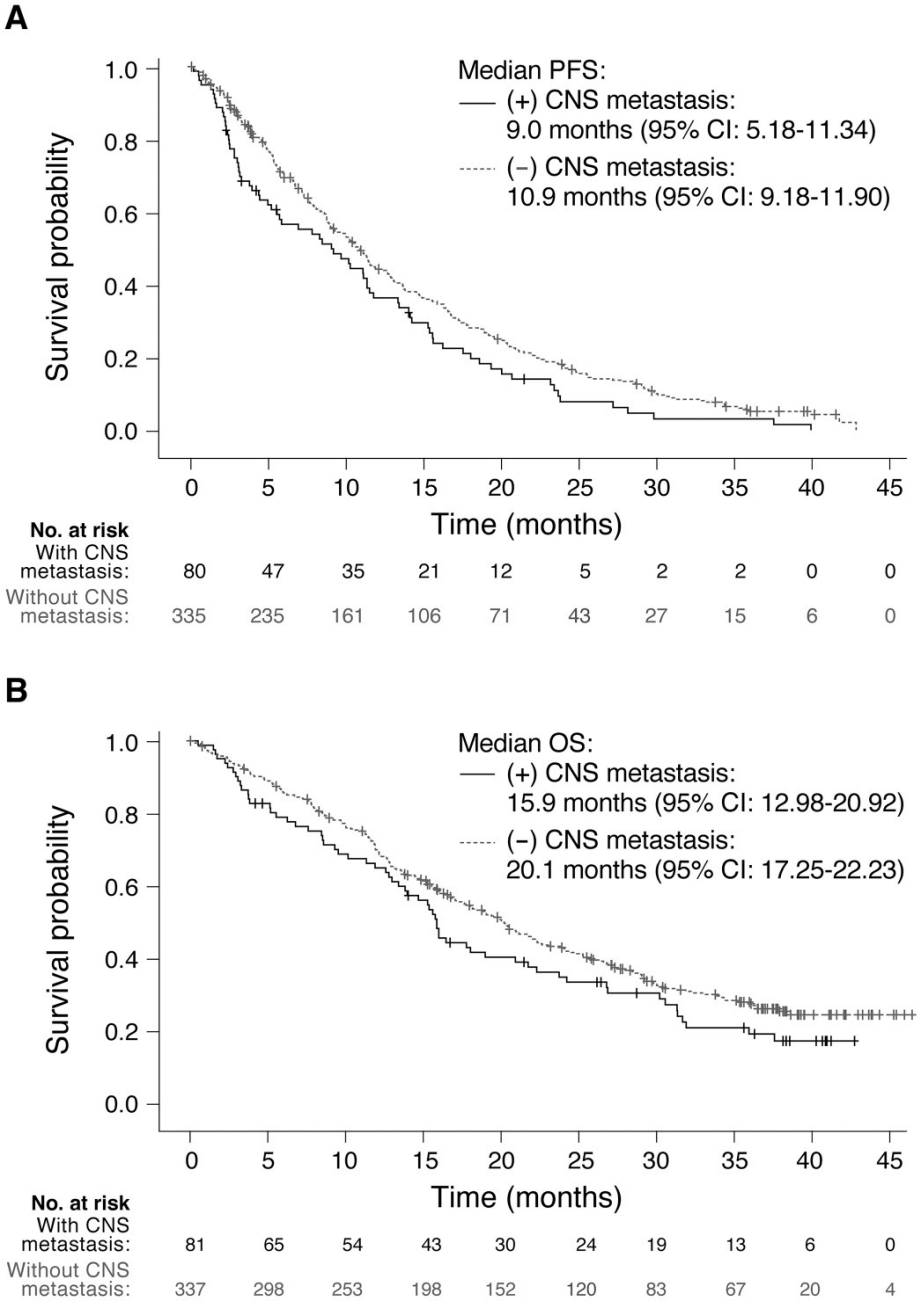
Kaplan‒Meier analysis of (A) PFS and (B) OS by CNS metastasis. CI, confidence interval; OS, overall survival; PFS, progression-free survival.

CNS metastasis was present in 18.91% of patients (Table 1). Further analysis and KM estimates of PFS and OS in this subgroup are shown in Fig 3A and 3B, respectively. Median PFS or OS did not significantly differ between patients with and without CNS metastasis ((9.0 [95% CI: 5.18–11.34] vs. 10.9 [95% CI: 9.18–11.90] months, respectively) and (15.9 [95% CI: 12.98–20.92] vs. 20.1 [95% CI: 17.25–22.23] months, respectively)).

#### Initial EGFR-TKIs prior to second-line Osimertinib

NSCLC treatments administered prior to second-line osimertinib are summarized in S5 Table. A total of 91 patients received osimertinib as second-line treatment. With respect to EGFR-TKIs administered prior to osimertinib, 24 (26.37%), 32 (35.16%) and 33 (36.26%) patients received afatinib, erlotinib and gefitinib, respectively. No significant differences in median PFS and OS were observed between different prior EGFR-TKI usages. The median PFS values in patients who had received prior afatinib, erlotinib and gefitinib were 11.2 (95% CI: 4.85–4.79), 10.5 (95% CI: 8.59–20.26) and 8.7 (95% CI: 7.21–16.79) months, respectively. The median OS in patients with prior afatinib, erlotinib and gefitinib were 18.0 (95% CI: 12.79–23.05), 23.7 (95% CI: 12.26,-), and 15.9 (95% CI: 11.57, 25.28) months, respectively (S6 Table and S3 Fig).

## Discussion

In this retrospective observational study of patients with EGFR T790M-positive locally advanced or metastatic NCSLC enrolled through an EAP in Taiwan, our findings align with previously published data on osimertinib and further support its clinical benefits and utility. Baseline characteristics were similar compared to AURA3 and ASTRIS studies, with one notable exception—our cohort was more heavily pretreated, with over 50% having received at least 3 lines of therapy prior to the initiation of osimertinib. Similarly, we included patients across the full spectrum of ECOG-PS, as opposed to only ECOG-PS of 0–1 and 0–2 in AURA3 and ASTRIS, respectively [16, 21]. Nevertheless, due to our study’s retrospective design, nearly half of the patients had an unknown ECOG-PS. ECOG-PS was previously suggested as an important prognostic indicator, with lower ECOG-PS consistently associated with longer PFS [17, 22]. As such, our cohort represented a population more advanced in their disease progression at baseline.

Nevertheless, participants in this study demonstrated a median PFS (10.5 months [95% CI: 8.95–11.41]) comparable to that of AURA3 (10.1 months [95% CI: 8.3–12.3]) and ASTRIS (11.1 months [95% CI: 11.0–12.0]) [16, 21]. The three studies had a difference in median PFS of only 12 to 30 days, likely attributable to differences in sample size and the aforementioned baseline characteristics. Pretreatment before osimertinib initiation in AURA3 participants involved primarily first- or second-generation EGFR-TKIs, whereas the inclusion of patients with prior chemotherapy in ASTRIS and our cohort represented a more diverse population reflective of real-world clinical practice. The slightly longer duration of PFS observed in these real-world studies suggests that prior chemotherapy may not affect the overall effectiveness of osimertinib. Similarly, the subsequent addition of chemotherapy after osimertinib monotherapy discontinuation also did not significantly influence our outcomes, as shown in the PFS and OS comparisons between the chemotherapy-osimertinib combination group and the osimertinib monotherapy group.

The effects of concurrent chemotherapy and osimertinib use in chemotherapy-naïve NSCLC patients have yet to be explored. A recent phase II trial in Japan (TAKUMI) examined the combination of carboplatin/pemetrexed with osimertinib in chemotherapy-naïve patients with T790M-positive stage IIIB/IV NSCLC [23]. In this prospective study, the addition of chemotherapy to osimertinib as a 2L treatment did not demonstrate any significant clinical benefit. Nevertheless, the safety profile was upheld in the trial results, leading the authors to conclude that a phase III trial investigating the use of osimertinib and chemotherapy in combination as a 1L treatment is warranted. Similarly, in our study, the addition of chemotherapy to osimertinib as subsequent therapy did not show a significant outcome, most likely due to the complex treatment history and relatively small number of patients in the subgroup analysis. The FLAURA trial outcomes have recently propelled osimertinib to 1L use with significant benefit in OS; however, acquired resistance to osimertinib is inevitable over time, with several complex mutations already identified [24–27]. The ongoing FLAURA2 trial investigating the addition of chemotherapy to 1L osimertinib for delaying the onset of acquired resistance will further elucidate this critical issue.

Notably, the median PFS and OS values in this cohort were found to be independent of prior lines of therapy despite heavy pretreatment. In AURA, the efficacy of osimertinib was comparable between second- and later-line use [13, 14]. Various real-world studies from Germany, France, and Japan also demonstrated findings similar to those observed in the Taiwan cohort [18–20]. A study examining the timing of T790M emergence and prescription of osimertinib as 2L treatment suggested that delayed initiation (>6 months) or the intercalation of other systemic therapies following rebiopsy may negatively impact PFS. Therefore, while later-line prescription of osimertinib may not detract from its overall efficacy, osimertinib’s targeted effectiveness against T790M-mediated acquired resistance suggests that prompt initiation once T790M emergence is confirmed improves outcomes [22].

For secondary endpoints, TTD was not assessed in the AURA trials but was found in this cohort and in ASTRIS to be 11.9 (95% CI: 10.49–13.11) and 13.5 (95% CI: 12.6–13.9) months, respectively [21]. In both studies, the main reason for osimertinib monotherapy discontinuation was PD. The difference in study population size between the Taiwan EAP and ASTRIS may have contributed to the disparity in median TTD. Interestingly, a longer median TTD concurrent with a longer median PFS was observed in ASTRIS when compared with the results in our study. Further investigation is needed to understand whether an increased duration of osimertinib monotherapy exposure would prolong the DFS.

The median OS of 19.0 months (95% CI: 16.30–20.95) in the Taiwan cohort was comparable to that of the France EAP cohort (20.5 months, 95% CI: 16.9–24.3), both slightly shorter than that of the AURA3 trial (26.8 months, 95% CI: 23.5–31.5) [15, 20]. This difference is likely a result of the difference between a strictly controlled clinical trial setting and real-world studies. Because OS data for ASTRIS, the largest real-world study to date, were immature at the time of publication, this hypothesis has yet to be confirmed. Notably, OS was defined as the time interval from the date of osimertinib initiation until the date of death from any cause in our study. As a result, the OS outcomes in our study not only demonstrated the effectiveness of osimertinib monotherapy but also included the effectiveness of osimertinib combined with subsequent therapies after PD. Thus, further investigation is needed to elucidate the following issues: 1) whether longer treatment duration with osimertinib prolongs OS, 2) whether the early introduction of combined treatment with osimertinib prolongs OS, and 3) the preferred subsequent therapy to be added to osimertinib that may prolong OS. Overall, regardless of the minor differences in PFS and TTD between studies as discussed above, the clinical benefits of osimertinib monotherapy were demonstrated in patients with EGFR T790M-positive NSCLC.

The best tumor response rates in this Taiwanese EAP cohort were encouraging, as over half of patients (53.91%) were deemed to have stable disease (SD) and 31.88% showed partial response (PR) to osimertinib. ORR (32.46%) fell short of the 71% rate reported in the AURA3 clinical trial but more closely approximates the 57.1% and 55.6% rates demonstrated in the ASTRIS and Japan real-world studies [16, 17, 21]. The DCR (86.38%) is broadly in line with that of AURA extension and AURA3 (90–93%) and consistent with the Japan real-world study (88.9%) [14, 16, 17].

Finally, in our subgroup analysis, no significant differences were observed in median PFS and OS when comparing patients with and without CNS metastases, which demonstrates consistent efficacy between the two groups. This finding further supports previous findings that osimertinib has superior penetration of the blood‒brain barrier, thereby mitigating between-group differences typically seen with other TKIs. The AURA3 and FLAURA studies have both demonstrated consistent efficacy regardless of the presence of CNS metastases at baseline [24, 28]. The FLAURA trials also revealed a reduced risk of CNS progression, higher CNS ORR, and lower cumulative incidence of new-onset CNS lesions with osimertinib vs. standard-of-care EGFR-TKIs in the CNS metastases cohort [24]. These results further support the superior role of 1L osimertinib in delaying CNS progression in advanced disease. We have also performed an additional analysis comparing PFS and OS between different initial EGFR-TKIs prior to second-line osimertinib because no such comparison has been reported in the AURA3 and FLAURA studies. The results revealed no significant differences, which indicates that the clinical effectiveness of osimertinib used in second-line therapy did not significantly vary across types of initial EGFR-TKIs.

This retrospective observational study has several limitations. First, the nature of such a study relies on convenience sampling. Our cohort notably consisted of patients with lower performance status and a more complex treatment history. Second, the evaluation of tumor response was based on individual investigator assessment, which may lack standardization. Thus, the potential for bias in tumor measurement-based outcomes such as PFS cannot be ruled out. In addition, real-world studies inevitably face the issue of missing data—PFS (*n* = 4), OS (1), and TTD (24)—which may affect the analysis of these endpoints and subsequent effectiveness of osimertinib treatment. However, because the proportion of missing data was relatively small (<6%) when compared to the FAS population (423 patients), the impact on this study was considered minimal. Last, while this study was not designed to assess the safety of osimertinib, it would have also contributed to a greater body of evidence toward its favorable clinical profile if safety data were collected for Taiwan patients.

## Conclusions

In summary, the efficacy of osimertinib demonstrated in clinical trials and observed in real-world studies is further substantiated by the results from this study. Clinical effectiveness and benefits of osimertinib are observed in pretreated Taiwanese patients with EGFR T790M-positive locally advanced or metastatic NSCLC in a real-world setting.

## Supporting information

S1 Fig Kaplan-Meier analysis of overall survival over time by plasma-based test and tissue biopsy. (DOCX)

S2 Fig Kaplan-Meier analysis of overall survival over time by previous lines of therapy. (DOCX)

S3 Fig Kaplan-Meier analysis Of (A) progression-free survival and (B) overall survival by EGFR TKI therapy prior to osimertinib used in second line. (DOCX)

S1 Table TNM staging when administered the first dose of osimertinib. (DOCX)

S2 Table EGFR mutation types, specimen types and testing platforms. (DOCX)

S3 Table Best tumor response rates. (DOCX)

S4 Table Subsequent treatment after discontinuation of osimertinib monotherapy. (DOCX)

S5 Table Summary of NSCLC therapy prior to osimertinib used in second line. (DOCX)

S6 Table Median progression-free and overall survival time by EGFR-TKI therapy prior to osimertinib used in second line. (DOCX)

S1 Checklist (DOCX)

S1 Data (XLSX)
