# Supplementary material for: Real-world osimertinib pretreatment experience in patients with epidermal growth factor receptor T790M mutation-positive locally advanced or metastatic non-small cell lung cancer
Source: PLoS One. 2024 May 16;19(5):e0303046. doi: 10.1371/journal.pone.0303046 (PMC11098304; doi:10.1371/journal.pone.0303046)
Supplement: S2 Fig — (DOCX) [file pone.0303046.s002.docx]

**S2 Fig. Kaplan-Meier Analysis of Overall Survival Over Time by Previous Lines of Therapy.**

CI, confidence interval; OS, overall survival.
