# Supplementary material for: Real-world osimertinib pretreatment experience in patients with epidermal growth factor receptor T790M mutation-positive locally advanced or metastatic non-small cell lung cancer
Source: PLoS One. 2024 May 16;19(5):e0303046. doi: 10.1371/journal.pone.0303046 (PMC11098304; doi:10.1371/journal.pone.0303046)

**S3 Fig. Kaplan-Meier Analysis Of (a) Progression-Free Survival and (b) Overall Survival by EGFR TKI Therapy Prior to Osimertinib Used in Second Line.**


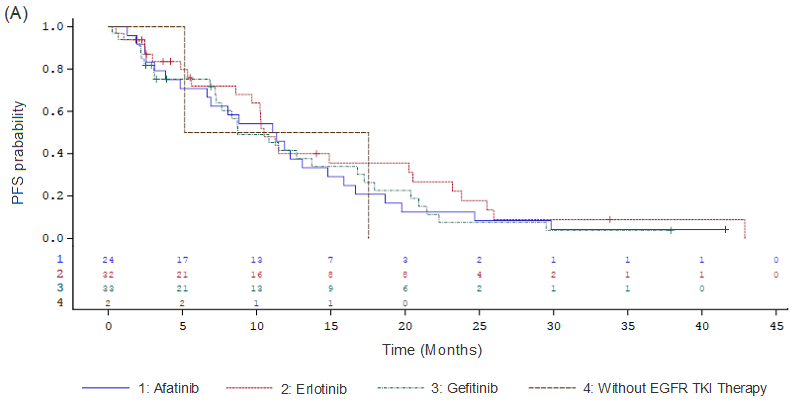


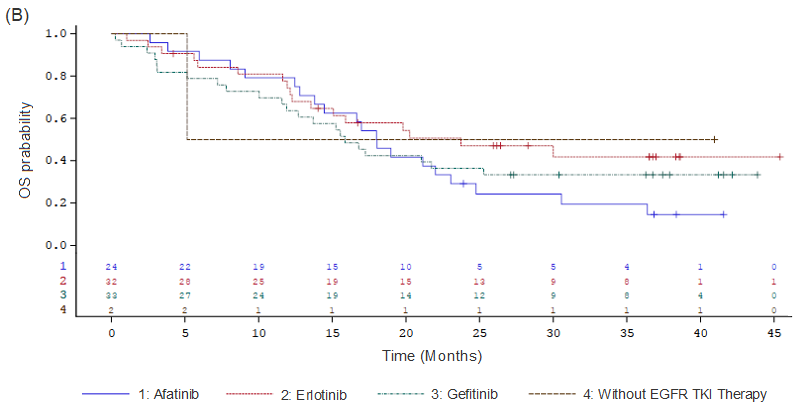

Supplement: S3 Fig — (DOCX) [file pone.0303046.s003.docx]
