# Supplementary material for: Real-world osimertinib pretreatment experience in patients with epidermal growth factor receptor T790M mutation-positive locally advanced or metastatic non-small cell lung cancer
Source: PLoS One. 2024 May 16;19(5):e0303046. doi: 10.1371/journal.pone.0303046 (PMC11098304; doi:10.1371/journal.pone.0303046)
Supplement: S1 Table — (DOCX) [file pone.0303046.s004.docx]

**S1 Table. TNM Staging when Administered the First Dose of Osimertinib**

| TNM staging | Total (n=423) |
| --- | --- |
| Not done | 198 (46.81 %) |
| T |  |
| 0 | 1 (0.24 %) |
| 1 | 11 (2.60 %) |
| 2 | 22 (5.20 %) |
| 3 | 15 (3.55 %) |
| 4 | 71 (16.78 %) |
| Unknown | 105 (24.82 %) |
| N |  |
| 0 | 15 (6.67 %) |
| 1 | 12 (5.33 %) |
| 2 | 30 (13.33 %) |
| 3 | 61 (27.11 %) |
| Unknown | 107 (47.56 %) |
| M |  |
| 0 | 5 (2.22 %) |
| 1 | 126 (56 %) |
| Unknown | 94 (41.78 %) |
