# Supplementary material for: Real-world osimertinib pretreatment experience in patients with epidermal growth factor receptor T790M mutation-positive locally advanced or metastatic non-small cell lung cancer
Source: PLoS One. 2024 May 16;19(5):e0303046. doi: 10.1371/journal.pone.0303046 (PMC11098304; doi:10.1371/journal.pone.0303046)
Supplement: S2 Table — (DOCX) [file pone.0303046.s005.docx]

**S2 Table. EGFR mutation types, specimen types and testing platforms.**

|  | **FAS Population (N = 423)** |
| --- | --- |
| **EGFR mutation type ^a^, n (%)**   \| exon 19 deletion \| \| --- \| \| exon 21 L858R \| \| exon 19 deletion, exon 20 insertion \| \| exon 19 deletion, exon 21 L858R \| \| exon 20 insertion \| \| exon 20 insertion, exon 21 L858R \| \| G719X \| \| G719X, S768I \| \| G719X, S768I, exon 20 insertion \| \| N771del insGY \| \| p.L861Q \| \| S768I, exon 21 L858R \| \| Unknown \|   **Sample** **Type/Test platform ^b^, n (%)**  **Plasma-based test** | \| 223 (52.72%) \| \| --- \| \| 157 (37.12%) \| \| 2 (0.47%) \| \| 1 (0.24%) \| \| 3 (0.71%) \| \| 1 (0.24%) \| \| 5 (1.18%) \| \| 1 (0.24%) \| \| 1 (0.24%) \| \| 1 (0.24%) \| \| 1 (0.24%) \| \| 2 (0.47%) \| \| 25 (5.91%) \|   **153 (36.34 %)** |
| Real-time PCR | 79 (18.76%) |
| MassARRAY | 37 (8.79%) |
| NGS | 4 (0.95%) |
| BEAMing / ddPCR | 9 (2.14%) |
| LDT | 7 (1.66%) |
| Unknown | 17 (4.04%) |
| **Tissue biopsy** | **187 (44.42 %)** |
| Real-time PCR | 89 (21.14%) |
| MassARRAY | 69 (16.39%) |
| BEAMing / ddPCR | 17 (4.04%) |
| LDT | 5 (1.19%) |
| Unknown | 7 (1.66%) |
| **Cytology** | **53 (12.59%)** |
| Real-time PCR | 16 (3.80%) |
| MassARRAY | 26 (6.18%) |
| BEAMing / ddPCR | 1 (0.24%) |
| LDT | 7 (1.66%) |
| Unknown | 3 (0.71%) |
| **Unknown sample type** | **28 (6.65%)** |

BEAMing, beads, emulsions, amplification, and magnetics; ddPCR, droplet digital polymerase chain reaction; EGFR, epidermal growth factor receptor; FAS, full analysis set; LDT, lab developed tests; NGS, next generation sequencing; PCR, polymerase chain reaction.

^a^EGFR mutation type FAS (Event [E] = 506); ^b^Sample type and test platform FAS (E = 421).
