# Supplementary material for: Real-world osimertinib pretreatment experience in patients with epidermal growth factor receptor T790M mutation-positive locally advanced or metastatic non-small cell lung cancer
Source: PLoS One. 2024 May 16;19(5):e0303046. doi: 10.1371/journal.pone.0303046 (PMC11098304; doi:10.1371/journal.pone.0303046)
Supplement: S3 Table — (DOCX) [file pone.0303046.s006.docx]

**S3 Table. Best tumor response rates.**

| \| **Best Tumor Response, n (%)** \| **FAS Population (N = 423)** \| \| --- \| --- \| | | |
| --- | --- | --- | --- | --- |
| Complete Response (CR)  Partial Response (PR) | 2 (0.58%)  110 (31.88 %) |  |
| Stable Disease (SD) | 186 (53.91 %) |  |
| Progressive Disease (PD) | 39 (11.30 %) |  |
| Not Evaluable (NE) | 8 (2.32 %) |  |
| Objective Response Rate (ORR) | 112 (32.46 %) |  |
| Disease Control Rate (DCR) | 298 (86.38 %) |  |
| Unknown | 78 (18.44 %) |  |

FAS, full analysis set.
