# Supplementary material for: Real-world osimertinib pretreatment experience in patients with epidermal growth factor receptor T790M mutation-positive locally advanced or metastatic non-small cell lung cancer
Source: PLoS One. 2024 May 16;19(5):e0303046. doi: 10.1371/journal.pone.0303046 (PMC11098304; doi:10.1371/journal.pone.0303046)
Supplement: S4 Table — (DOCX) [file pone.0303046.s007.docx]

**S4 Table. Subsequent Treatment After Discontinuation of Osimertinib Monotherapy.**

| **Subsequent Therapy** | **FAS Population**  **(N = 182; E = 370)** |
| --- | --- |
| **Subsequent combination treatment with osimertinib, n (%)**  **Chemotherapy**  Platinum-based chemotherapy  Other chemotherapy  **Vascular endothelial growth factor (VEGF)**  VEGF alone  VEGF combination  **Immunotherapy (IO)**  IO alone  IO combination  **EGFR-TKI therapy**  EGFR-TKI alone  EGFR-TKI combination  **Others**^a^  **Subsequent treatment without osimertinib, n (%)**  **Chemotherapy**  Platinum-based chemotherapy  Other chemotherapy  **Vascular endothelial growth factor (VEGF)**  VEGF alone  VEGF combination  **Immunotherapy (IO)**  IO alone  IO combination  **EGFR-TKI therapy**  EGFR-TKI alone  EGFR-TKI combination  **Others**^a^ | 30 (8.11%)  134 (36.22%)  16 (4.32%)  12 (3.24%)  3 (0.81%)  9 (2.43%)  9 (2.43%)  1 (0.27%)  26 (7.03%)  26 (7.03%)  99 (26.76%)  4 (1.08%)  19 (5.14%)  9 (2.43%)  6 (1.62%)  13 (3.51%)  3 (0.81%)  17 (4.59%) |

E, events; EGFR-TKI, epidermal growth factor receptor sensitizing mutation tyrosine kinase inhibitors; FAS, full analysis set; n, number; N, total number in population.

^a^Others include brigatinib, irinotecan, cetuximab, DS 1205, etoposide, methotrexate, savolitinib, tegafur, topotecan, ribociclib.
