# Supplementary material for: Real-world osimertinib pretreatment experience in patients with epidermal growth factor receptor T790M mutation-positive locally advanced or metastatic non-small cell lung cancer
Source: PLoS One. 2024 May 16;19(5):e0303046. doi: 10.1371/journal.pone.0303046 (PMC11098304; doi:10.1371/journal.pone.0303046)
Supplement: S5 Table — (DOCX) [file pone.0303046.s008.docx]

**S5** **Table.** **Summary of NSCLC Therapy Prior to Osimertinib Used in Second Line.**

| **NSCLC therapy, n (%)** | **FAS Population (N = 91)** |
| --- | --- |
| **EGFR-TKI therapy** |  |
| Afatinib | 24 (26.37 %) |
| Erlotinib | 32 (35.16 %) |
| Gefitinib | 33 (36.26 %) |
| **Chemotherapy** |  |
| Platinum- or taxane-based therapy^a^ | 1 (1.10 %) |
| Other chemotherapy | 2 (2.20 %) |
| **Other systemic therapy** |  |
| Bevacizumab | 1 (1.10 %) |
| Nivolumab | 1 (1.10 %) |
| **Radiation therapy for brain metastasis** |  |
| Radiation alone | 4 (4.40 %) |
| Radiation combined with systemic therapy^b^ | 8 (8.79 %) |
| **Combined therapy** |  |
| EGFR-TKI combined with other systemic therapy | 2 (2.20 %) |

EGFR-TKI, epidermal growth factor receptor sensitizing mutation tyrosine kinase inhibitors; n, number; N, total.

aPlatinum-based therapies include carboplatin and cisplatin. Taxane-based therapies include docetaxel, nab-paclitaxel, and paclitaxel. The combination of platinum- or taxane-based therapy with other chemotherapy was included in this category.

^b^Included radiation therapy for brain metastasis combined with EGFR-TKI, chemotherapy, or other systemic therapy.
