# Supplementary material for: Real-world osimertinib pretreatment experience in patients with epidermal growth factor receptor T790M mutation-positive locally advanced or metastatic non-small cell lung cancer
Source: PLoS One. 2024 May 16;19(5):e0303046. doi: 10.1371/journal.pone.0303046 (PMC11098304; doi:10.1371/journal.pone.0303046)
Supplement: S6 Table — (DOCX) [file pone.0303046.s009.docx]

**S6 Table.** **Median Progression-Free and Overall Survival Time by EGFR-TKI Therapy Prior to Osimertinib Used in Second Line.**

|  | | | | | |
| --- | --- | --- | --- | --- | --- |
|  | **Total (N = 91)** | | | | |
|  | **Number of subjects** | **Progression, N (%)** | **Death, N (%)** | **Median (95% CI) (months)** | **p-value** |
| **Progression free survival** |  |  |  |  | 0.7531 |
| Afatinib | 24 | 21 (87.50) | 2 (8.33) | 11.2 (4.85, 14.79) |  |
| Erlotinib | 32 | 20 (62.50) | 4 (12.50) | 10.5 (8.59, 20.26) |  |
| Gefitinib | 33 | 18 (54.55) | 9 (27.27) | 8.7 (7.21, 16.79) |  |
| Without EGFR TKI | 2 | 1 (50.00) | 1 (50.00) | 11.3 (5.15, -) |  |
| **Overall survival** |  |  |  |  | 0.5668 |
| Afatinib | 24 | - | 20 (83.33) | 18.0 (12.79, 23.05) |  |
| Erlotinib | 32 | - | 17 (53.13) | 23.7 (12.26, -) |  |
| Gefitinib | 33 | - | 22 (66.67) | 15.9 (11.57, 25.28) |  |
| Without EGFR TKI | 2 | - | 1 (50.00) | - (5.15, -) |  |
| CI, confidence interval; n, number; N, total number in population. | | | | | |
